# The Encapsulation of Hemagglutinin in Protein Bodies Achieves a Stronger Immune Response in Mice than the Soluble Antigen

**DOI:** 10.3389/fpls.2016.00142

**Published:** 2016-02-16

**Authors:** Anna Hofbauer, Stanislav Melnik, Marc Tschofen, Elsa Arcalis, Hoang T. Phan, Ulrike Gresch, Johannes Lampel, Udo Conrad, Eva Stoger

**Affiliations:** ^1^Department of Applied Genetics and Cell Biology, University of Natural Resources and Life SciencesVienna, Austria; ^2^Department of Molecular Genetics, Leibniz Institute of Plant Genetics and Crop Plant ResearchGatersleben, Germany

**Keywords:** protein bodies, molecular farming, subcellular targeting, recombinant protein, recombinant vaccine

## Abstract

Zein is a water-insoluble polymer from maize seeds that has been widely used to produce carrier particles for the delivery of therapeutic molecules. We encapsulated a recombinant model vaccine antigen in newly formed zein bodies *in planta* by generating a fusion construct comprising the ectodomain of hemagglutinin subtype 5 and the N-terminal part of γ-zein. The chimeric protein was transiently produced in tobacco leaves, and H5-containing protein bodies (PBs) were used to immunize mice. An immune response was achieved in all mice treated with H5-zein, even at low doses. The fusion to zein markedly enhanced the IgG response compared the soluble H5 control, and the effect was similar to a commercial adjuvant. The co-administration of adjuvants with the H5-zein bodies did not enhance the immune response any further, suggesting that the zein portion itself mediates an adjuvant effect. While the zein portion used to induce protein body formation was only weakly immunogenic, our results indicate that zein-induced PBs are promising production and delivery vehicles for subunit vaccines.

## Introduction

Polymers are widely used as carrier biomaterials for the delivery of therapeutic molecules (Petros and DeSimone, 2010). In particular, biopolymer-based nanoparticles have proven suitable for clinical applications due to their biocompatibility and biodegradability (Panyam and Labhasetwar, 2003; Nitta and Numata, 2013). A variety of materials and preparation methods have been developed for application-specific properties in terms of particle shape, surface charge, and surface features (Petros and DeSimone, 2010). Among the protein-based biopolymers, those derived from natural proteins such as silk, collagen, elastin, and fibronectin have been studied in detail (Ruszczak and Friess, 2003; Daamen et al., 2007; Lammel et al., 2010; Nitta and Numata, 2013).

Zein, a protein-based polymer found in maize seeds, has been widely used as a carrier because of favorable properties such as biocompatibility, insolubility and low water uptake, mechanical and chemical stability, and its propensity to form coatings and microparticles (Liu et al., 2005; Lai and Guo, 2011; Wang et al., 2011; Lau et al., 2013). Zein is also generally regarded as safe (GRAS) for food use and resists digestion, making it particularly suitable as an encapsulation polymer for oral drugs (Hurtado-Lopez and Murdan, 2006b; Gong et al., 2011; Lau et al., 2013; Zou and Gu, 2013; Ahmed et al., 2015). The intravenous delivery of drug-loaded zein-based microparticles has also been investigated as a means to achieve long-acting effects such as the slow and sustained release of pharmaceutical compounds (Lai and Guo, 2011) and more efficient drug delivery to cancer cells (Lin et al., 2011; Podaralla et al., 2012; Lohcharoenkal et al., 2014). Zein-based microspheres may also provide adjuvant effects when used as vaccine carriers (Hurtado-Lopez and Murdan, 2006a).

The *in vitro* loading of zein-based microparticles with drugs usually involves spray or freeze drying or liquid–liquid dispersion methods (Zhong and Jin, 2009; Podaralla and Perumal, 2010; Podaralla et al., 2012; Zou and Gu, 2013). These technical processes are expensive and can affect the activity of the encapsulated agent, e.g., the high temperatures required for spray drying are incompatible with many pharmaceutical proteins.

It is therefore appealing to use plants to achieve microencapsulation *in vivo* by directly incorporating recombinant proteins into naturally occurring protein storage organelles such as zein bodies (Hofbauer and Stoger, 2013). Endogenous protein storage organelles are usually found in plant seeds, and zein-like prolamins are characteristic features of cereal endosperm cells. In production systems based on cereal seeds, the recombinant protein is often targeted to accumulate in prolamin-containing storage organelles that provide a protective environment, even offering some resistance against proteolytic digestion in simulated gastric fluids (Takaiwa et al., 2015).

Instead of using the natural prolamin bodies that are formed in rice, wheat, maize or barley endosperm, it is also possible to fuse the recombinant protein to assembly sequences that induce analogous structures in tissues such as leaves, which usually lack protein storage organelles. This ectopic protein body technology bypasses the longer generation time required to produce cereal seeds while still offering the advantages of natural bioencapsulation. Sequences that share the ability to trigger the formation of ectopic protein bodies (PBs) include those derived from cereal prolamins, synthetic elastin-like peptides (ELPs) and fungal hydrophobins (Floss et al., 2008; Conley et al., 2009; Torrent et al., 2009b; Gutierrez et al., 2013; Shigemitsu et al., 2013).

One of the most widely used assembly sequences comprises the N-terminal part of the mature 27 kD γ-zein protein, a member of the major prolamin-type storage protein family in maize (Shewry and Halford, 2002). Unlike other assembly sequences, it not only induces the formation of PBs but also acts as a retention sequence that stops fusion proteins from leaving the endoplasmic reticulum (ER). Consequently, the induced PBs bud from the ER as distinct round structures, underscoring the intrinsic compartment-forming properties of the zein sequence in the absence of tissue-specific factors (Mainieri et al., 2004; Llop-Tous et al., 2010). The N-terminal sequence of the 27 kD γ-zein protein comprises two cysteine residues downstream of the signal peptide, a repeated proline-rich domain forming an amphipathic helix, and a third section that includes four additional cysteine residues (Geli et al., 1994). Several reports have confirmed that zein-derived sequences induce ectopic PBs when appended to either the N-terminus or the C-terminus of diverse recombinant proteins, including phaseolin (Mainieri et al., 2004), enhanced cyan fluorescent protein (Llop-Tous et al., 2010), xylanase (Llop-Tous et al., 2011), DsRed (Joseph et al., 2012), and the *Human papillomavirus* E7 protein (Whitehead et al., 2014). Moreover, the ability to induce PBs appears to be almost entirely intrinsic and independent of other host-specific factors, thus allowing the formation of ectopic PBs in fungal, insect and mammalian cells (Torrent et al., 2009a), and the budding of PBs from ectopic membranes such as the plastid envelope, when combined with alternative subcellular targeting strategies (Hofbauer et al., 2014).

Hemagglutinin is an abundant type I integral membrane glycoprotein found on the envelope of influenza viruses and it has been widely used in influenza vaccine development and as a model antigen. The precursor protein HA0 yields two chains, i.e., HA1 (∼36 kDa) and HA2 (∼28 kDa), following cleavage at the motif Q/E-X-R. Infectivity requires both chains to be glycosylated, and also relies on the cleavage of hemagglutinin by a protease at multiple arginine residues. (Klenk and Garten, 1994; Hulse et al., 2004). The cleavage products are then covalently linked by a disulfide bond and these HA1/HA2 units form non-covalent homotrimers (Wiley et al., 1977).

A transmembrane domain is found near the C-terminus of HA2. The three-dimensional structure of hemagglutinin reveals two domains: a stem, responsible for membrane anchoring (part of HA1 and all of HA2), and a globular head (only HA1), bearing the sialic acid receptor binding domains (RBDs) (Wilson et al., 1981). In this study we generated a fusion construct comprising the ectodomain of hemagglutinin subtype 5 and the N-terminal part of γ-zein (amino acids 4–93) in order to induce the storage of the recombinant fusion protein inside newly formed PBs. The chimeric protein was transiently produced in tobacco leaves and H5-containing PBs were used to immunize mice. The resulting immune response was compared to that of control groups administered with the soluble H5 antigen, with or without adjuvant.

## Materials and Methods

### Vector Constructs

All cloning steps were carried out using the binary vector pTRA, a derivative of pPAM (GenBank AY027531). The sequence corresponding to the H5 ectodomain (amino acids 17–520) of hemagglutinin from the A/Hatay/2004/(H5N1) influenza strain (GenBank Q5QQ29) was amplified as described (Phan et al., 2014) and a plant codon-optimized signal peptide sequence derived from a murine antibody was added to the N-terminus to direct the protein into the secretory pathway (Vaquero et al., 1999). Amino acids 4–93 of the mature 27 kD γ-zein protein (lacking the signal peptide) were joined to the C-terminus via a (GGGS)_2_ linker as previously described for the phaseolin fusion construct zeolin (Mainieri et al., 2004). A His_6_-tag was added to the C-terminus for detection. The final expression vector “H5-Zein” was produced by transferring this coding sequence to the pTRA vector between the *Tobacco etch virus* (TEV) 5′-untranslated region and the *Cauliflower mosaic virus* (CaMV) 35S terminator. The expression construct was thus placed under the control of the CaMV 35S promoter with a duplicated transcriptional enhancer. An analogous construct comprising only the H5 ectodomain, a His_6_-tag and a C-terminal KDEL sequence was used to produce the soluble H5 antigen.

### Plant Material

Tobacco (*Nicotiana benthamiana*) plants were cultivated in soil in a growth chamber with a 16-h photoperiod, 26/16°C day/night temperatures and 70% relative humidity for 2 months.

### Agroinfiltration of Tobacco Leaves

The expression constructs were transferred by electroporation into competent *Agrobacterium tumefaciens* (GV3101) cells. The bacteria were kept as a glycerol stock and used to inoculate 5-ml aliquots of YEB medium containing 25 mg/l kanamycin, 25 mg/l rifampicin, and 50 mg/l carbenicillin. The cultures were incubated for 2 days at 28°C, shaking at 180 rpm. Each culture was mixed 1:1 with a culture containing a silencing inhibitor (HcPro) before adjusting with 2x infiltration medium (100 g/l sucrose, 3.6 g/l glucose, 8.6 g/l MS salts, pH 5.6) to an OD_600_ of ∼1.0. After adding 200 μM acetosyringone, *N. benthamiana* leaves were infiltrated using a syringe (for small-scale expression) or vacuum (for large-scale expression). Young plants were completely submerged in the suspension and vacuum was applied for 2 min. The infiltrated leaves were harvested 7 days post-infiltration (DPI).

### Protein Purification

#### Soluble H5: Immobilized Metal Affinity Chromatography (IMAC)

Frozen leaf powder was mixed at a ratio of 1:2 (w/w) with cold lysis buffer (50 mM sodium phosphate buffer, pH 8.0, 300 mM NaCl, 5 mM imidazole, 0.5 mM PMSF) and sonicated briefly to induce further cell lysis. After 2 h, the suspension was centrifuged at 9000 rpm for 20 min and the supernatant was passed through a 1-μm filter. The pH was re-adjusted to 8.0 and the suspension was centrifuged as above. The supernatant was passed through a 0.45-μm filter before mixing with Ni-IDA IMAC resin (BioRad, Munich, Germany). Approximately 2 ml of 50% resin suspension was added per 50 ml supernatant. After incubation for 1 h, the resin was loaded onto a column and washed with eight volumes of wash buffer (50 mM sodium phosphate buffer, pH 8.0, 300 mM NaCl, 10 mM imidazole). The protein was eluted with elution buffer (50 mM sodium phosphate buffer, pH 8.0, 300 mM NaCl, 250 mM imidazole). The amount of protein in each fraction was determined using the Bradford assay before immunoblot analysis.

#### Protein Bodies (Density Gradient Centrifugation)

The frozen leaf powder was mixed 1:1 (w/v) with extraction buffer (10 mM Tris-HCl, 0.4 M sucrose, pH 7.5) and incubated overnight at 4°C with constant shaking. The homogenate was passed through two layers of miracloth to remove solid debris and then loaded on a discontinuous sucrose gradient (3, 2.5, 2, 1.5, and 1 M in 10 mM Tris-HCl, pH 7.5). This preparation was centrifuged at 30,000 rpm and 4°C for 3 h in a Beckman ultracentrifuge (SW 41Ti or SW32TI rotor). After separation, 500-μl fractions were collected for analysis by SDS-PAGE and immunoblotting.

### Protein PAGE and Immunoblot Analysis

Infiltrated leaves (7 DPI) were harvested and ground in liquid nitrogen to a fine powder. We then extracted 60 mg of leaf powder in 200 μl buffer K (62.5 mM Tris, pH 7.4, 10% glycine, 5% 2-mercaptoethanol, 2% SDS, 8 M urea). Ten microliter of the extract were mixed with loading buffer and boiled for 10 min before loading. Samples collected from the density gradient and IMAC procedures were mixed with 5x loading buffer, boiled at 100°C for 10 min and separated by reducing SDS-PAGE (12% polyacrylamide gel, 200 V for 90 min). Gels were stained with Coomassie Blue or transferred to a nitrocellulose membrane. The membrane was blocked with 5% (w/v) skimmed milk in phosphate buffered saline (PBS) for 1 h and then incubated with a mouse anti-poly-histidine antibody (Sigma-Aldrich Chemie GmbH, Germany) at room temperature for 2 h, diluted 1:10000. The blot was washed three times in PBS plus 0.05% Tween-20 (PBST) and then incubated for 1 h with the secondary anti-mouse alkaline phosphatase-conjugated antibody (diluted 1:5000). The membrane was washed another three times with PBST and the signal was detected using the NBT/BCIP system. For quantitation, the samples were compared to serial dilutions of a His_6_-tagged standard protein, and the images were analyzed using BioRad Image Lab v5.1.

### Fluorescence Microscopy

Infiltrated leaves were cut into small pieces with a razor blade and fixed in 4% (w/v) paraformaldehyde plus 0.5% (v/v) glutaraldehyde in 0.1 M phosphate buffer (pH 7.4) at 4°C overnight. For immunolocalization by confocal microscopy, vibratome sections were mounted on a glass slide, blocked with 5% (w/v) bovine serum albumin (BSA) in 0.1 M phosphate buffer (pH 7.4) and incubated with a polyclonal antibody against 27 kD γ-zein. The samples were then incubated with an AlexaFluor^®^488-conjugated secondary antibody and observed under a Leica SP5 confocal laser scanning microscope (CLSM).

### Immunization of Mice

Male BL6 C57/Bacl6J mice (6–8 weeks old) obtained from Charles River Laboratories, Research Models and Services were assigned to seven groups (*n* = 10). Immunization was carried out by the subcutaneous injection of 150 or 300 ng of H5-zein either with or without Freund’s adjuvant (1:1) (Difco Laboratories, Detroit, Michigan). For the primary immunization, complete Freund’s adjuvant was used where indicated. Booster immunizations consisted of two additional injections of the same antigen (with or without incomplete Freund’s adjuvant). As controls, one group received PBS with Freund’s adjuvant only, and two groups were injected three times with soluble H5 (15 μg), with or without adjuvant. After the third immunization, the mice were retro-orbitally bled and serum samples were collected for individual testing. A second set of blood samples was taken 8 weeks after the primary immunization and the mice were sacrificed immediately afterward.

The animal experiments were approved by the Landesverwaltungsamt Sachsen-Anhalt, Halle/Saale, Referat Verbraucherschutz, Veterinärangelegenheiten and by the Landkreis Harz, Amt für Veterinärwesen und Lebensmittelüberwachung, Halberstadt. All animals received humane care according to the requirements of the German Animal Welfare Act, §8 Abs. 1.

### IgG Quantitation by ELISA

The wells of a flat-bottom microtiter plate were coated with 0.2 μg per well of the antigen (purified recombinant H5 or zein (Sigma–Aldrich Chemie GmbH, Germany)). Then 100 μl of diluted serum (1:250 in PBS with 3% BSA) was added to each well and incubated at room temperature for 1.5 h. Rabbit anti-mouse IgG alkaline phosphatase-conjugated antibody (diluted 1:2000 in PBST) was used for detection. The IgG titer was determined by adding immune serum samples as serial dilutions starting at 1:1000. Curve fitting by five-parameter logistic regression was used to calculate the endpoint titer for each mouse. End-point titers were determined as the reciprocal highest serum dilutions that produced mean optical density values two-fold greater than the geometric mean of those from the negative control (injected with PBS) sera. The statistical significance was determined using Student’s *t*-test (^∗∗^*p* < 0.01; ^∗^*p* < 0.1).

Selected ELISA experiments were carried out using recombinant H5 purified via an additional size exclusion chromatography step. The same results were obtained, indicating that the H5 preparation purified via IMAC was sufficiently pure for coating, and did not contain significant amounts of immunoreactive impurities.

### Hemagglutination Inhibition (HI) Test

HI tests were carried out as described by Phan et al. (2013). Briefly, a 25-μl aliquot of murine serum was mixed with 25 μl PBS and added to the first well of a V-bottom microtiter plate. Twofold serial dilutions were prepared across the row of 12 wells. Aliquots (25 μl) containing 4HAU of inactivated virus [A/swan/Germany/R65/2006(H5N1)] were added to each well and incubated for 30 min at room temperature. We then pipetted 25 μl of a 1% red blood cells (RBCs) suspension into each well and the plate was again incubated for 30 min at room temperature. The HI titer was defined as the reciprocal of the highest serum dilution that achieved the complete inhibition of hemagglutination.

## Results

### Hemagglutinin-Zein Fusions form PBs in *N. benthamiana* Leaves

**FIGURE 1:**
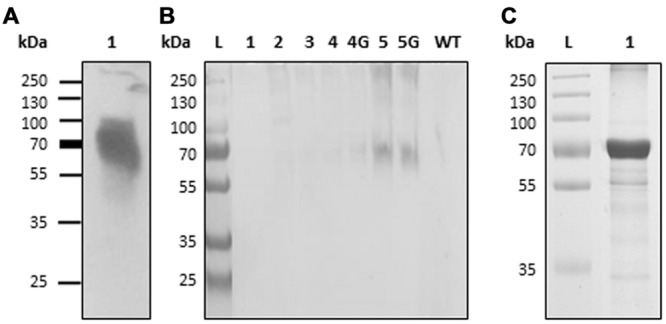
**Production and isolation of H5-zein and H5. (A)** Immunoblot analysis of a leaf extract containing H5-zein. **(B)** Immunoblot of H5-zein samples following the sucrose density gradient separation of leaf homogenates. Lanes 1–5: Selected samples from different density steps along the gradient are shown (lane 1: sample from the top of the gradient; lane 5: pellet. 4G and 5G are the same fractions as 4 and 5, but obtained from a different experiment). An antibody specific for the His_6_ tag was used for detection. **(C)** H5 after IMAC purification (3 μg were leaded on the gel and stained with Coomassie). wt, wild type; L, molecular weight ladder in kDa.

**FIGURE 2:**
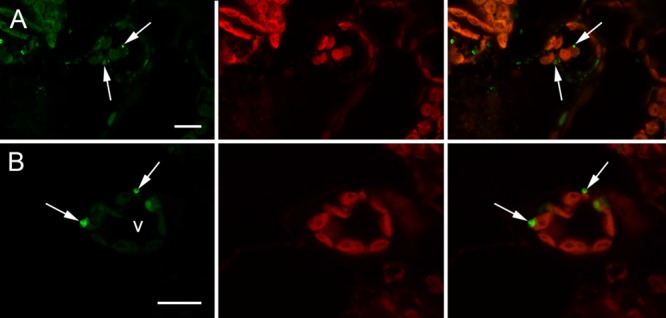
**Immunolocalization of H5-zein in *N. benthamiana* leaves by confocal microscopy. (A)** Abundant H5-zein protein bodies (PBs) can be observed in mesophyll cells (arrows). **(B)** H5-zein PBs are 1–2 μm in diameter and can be found in the cytoplasm between the chloroplasts (arrows). The left panel shows the detection of the fusion protein. The middle panel shows autofluorescence of the chloroplasts. The right panel shows the overlay pictures. Abbreviations: v, vacuole. Bars = 10 μm.

Expression vector “H5-Zein” containing the sequence corresponding to the H5 ectodomain of hemagglutinin, fused to amino acids 4–93 of the mature 27 kD γ-zein protein, was introduced into *N. benthamiana* leaves by agroinfiltration. Immunoblot analysis of extracts from the infiltrated leaves 7 DPI revealed the presence of a band corresponding to the fusion protein (**Figure 1A**). The higher than predicted molecular mass probably reflected the glycosylation of H5, as previously reported (Phan et al., 2014). The fusion of H5 to zein resulted in the formation of PBs, whose presence was confirmed by immunofluorescence microscopy. All labeling was concentrated in the PBs, whereas no signal was detected in the ER lumen or in the apoplast (**Figure 2**). This result was confirmed by the density step gradient centrifugation of leaf homogenates (**Figure 1B**). The gradient fractions were collected and tested by immunoblot analysis. No recombinant fusion protein was found in fractions from the top of the gradient, a small amount was present in the highest density fractions, and the majority of the fusion protein was found in the pellet (**Figure 1B**), similar to results reported with another zein fusion protein that forms PBs (Whitehead et al., 2014). Sucrose was removed by pooling the selected fractions and resuspending them in 10 mM Tris (pH 7.5) before centrifuging them under the same conditions as above. The supernatant was removed and the pellet was re-suspended in sterile PBS. The protein suspension was stored at –20°C prior to the immunization experiments. A total of ∼120 μg H5-zein was recovered from 300 g of fresh infiltrated leaves. Soluble H5 lacking the zein fusion was expressed as a control and purified by IMAC as previously described (Phan et al., 2013). We recovered 1 mg of H5 from 500 g of fresh infiltrated leaves, and this was used as a positive control for immunization (**Figure 1C**).

### H5-Zein PBs Elicit an Immune Response in Mice

**FIGURE 3:**
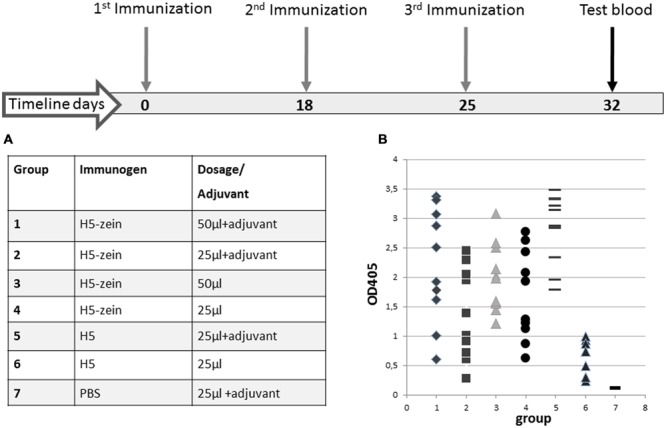
**Immunization timeline and IgG responses in the seven treatment groups. (A)** All mice were injected with a primary dose of H5-zein (6 μg/ml) or H5 (0.6 mg/ml), both in sterile PBS, with or without Freund’s complete adjuvant. For the second and third injections, the adjuvant was switched to Freund’s incomplete adjuvant. **(B)** ELISA analysis of the anti-H5 IgG response following the third immunization. A single dot represents the ELISA result from a single serum sample. Each treatment group comprised 10 mice.

The H5-zein protein body suspension and the soluble H5 antigen were each used to immunize mice (**Figure 3**). The mice were allocated to seven groups (*n* = 10 per group) and immunization was carried out by the subcutaneous injection of 150 or 300 ng of H5-zein either with or without Freund’s adjuvant. This low dosage of H5-zein bodies was chosen to confirm the hypothesis that particulate antigens are effective in small amounts. As controls, two groups were injected three times with soluble H5 (15 μg, a dose previously confirmed to provoke a strong humoral immune response), one with and one without adjuvant.

The plant-derived H5-zein protein body suspension was shown to elicit an IgG response in 100% of the animals, even at low doses. Interestingly, the addition of an adjuvant to the H5-zein bodies did not cause a significantly stronger immune response (**Figure 3**, groups 1 vs. 3 and 2 vs. 4), whereas the adjuvant had a significant impact in the control groups receiving soluble H5 (**Figure 3**, groups 5 vs. 6).

### The Zein Fusion Component is Only Weakly Immunogenic but has a Significant Adjuvant Activity

**FIGURE 4:**
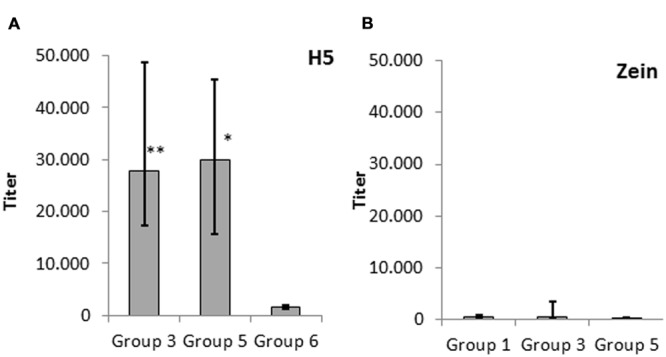
**Serum IgG response against **(A)** H5 and **(B)** zein.** Data represent the median endpoint titer per group, whereas the lower and upper ends of the error bars represent the first and third quartiles, respectively. **(A)** Statistical analysis to compare group 3 (H5-zein without adjuvant) and group 5 (H5 with adjuvant) vs. group 6 (H5 without adjuvant). **(B)** Statistical analysis to compare group 1 (H5-zein with adjuvant) and group 3 (H5-zein without adjuvant) vs. group 5 (H5 with adjuvant). Each treatment group comprised 10 mice. Statistical significance was determined by Student’s *t*-test (^∗∗^*p* < 0.01; ^∗^*p* < 0.1; no asterisk indicates *p* > 0.1).

To confirm the observations summarized above, we carried out an in-depth comparison of IgG titers of groups 3, 5, and 6. The IgG response elicited by H5-zein without adjuvant (group 3) was comparable to that achieved by injecting soluble H5 combined with an adjuvant (group 5). In contrast, the administration of soluble H5 without adjuvant elicited a minimal IgG response (**Figure 4A**). This suggested that the zein component and/or the particulate nature of the protein body act as an adjuvant.

To determine whether the zein portion fused to H5 has intrinsic immunogenic properties, we investigated the IgG response directed against zein by comparing the IgG response in groups 1 (H5-zein with adjuvant), 3 (H5-zein without adjuvant), and 5 (soluble H5 with adjuvant). Although we generally detected low IgG titers, 30% of the animals in groups 1 and 3 showed a clearly detectable IgG response against zein (**Figure 4B**). Overall the immune response in the treatment groups was not significantly different to that of the control group (*p* > 0.1).

### HI Antibody Titers are Insignificant

All mice vaccinated with H5-zein PBs showed an immunological response so we carried out HI tests on the serum from each mouse to determine whether the induced antibodies were potentially capable of neutralizing the virus. Because of the unavailability of the A/Hatay/2004(H5N1) virus in an inactivated form, the heterologous inactivated virus strain A/swan/Germany/R65/2006(H5N1) was used for the HI assay. The deduced hemagglutinin amino acid sequence similarity of both strains is 96%, and it was previously shown that HI titres against inactivated virus A/swan/Germany/R65/2006 (H5N1) could be measured in sera from mice vaccinated with trimeric HA derived from the HA sequence corresponding to the A/Hatay/2004(H5N1) virus (Phan et al., 2013). The HI assay results indicated that the HI antibody titers were either below or marginally above the detection limit in all treatment groups (**Table 1**).

**Table 1:** HI titers against inactivated virus [A/swan/Germany/R65/2006(H5N1)].

Treatment group	Geometric mean titer
1 (H5-zein + adjuvant)	6,06
3 (H5-zein)	4,29
6 (H5)	3,4
7 (PBS)	7,46

## Discussion

The expression of recombinant proteins in plants is an attractive strategy reflecting the versatility, safety, scalability, and economy of plant-based production platforms (Rybicki et al., 2013; Stoger et al., 2014). Plants also offer the possibility to accumulate recombinant pharmaceutical proteins within endogenous or ectopic protein storage organelles, which can either be derived directly from the ER or represent protein storage vacuoles (Khan et al., 2012). Here, we successfully induced the formation of ectopic PBs by fusing the H5 ectodomain of hemagglutinin to the N-terminal sequence of γ-zein. Previous studies have shown that the biogenesis of PBs by zein is influenced by the fusion partner, and that not all fusion proteins support the efficient formation of PBs. For example, phaseolin induces the efficient formation of zeolin PBs when fused to the N-terminal sequence of γ-zein (Mainieri et al., 2004). However, PBs were not formed when the *Human immunodeficiency virus* Nef antigen was fused to the same γ-zein sequence, but protein body formation was possible again when Nef was fused to the entire chimeric protein zeolin (de Virgilio et al., 2008).

The induction of H5-containing protein aggregates in plants has also been achieved by fusing the antigen to hydrophobin and ELPs (Phan et al., 2014). The H5-ELP PBs were approximately 800 nm in diameter whereas the H5-hydrophobin PBs were substantially smaller, with an average diameter of 250 nm. The H5-zein PBs reported herein were larger, with a diameter of 1–2 μm, which is similar to the average size of endogenous zein bodies found in maize endosperm (Lending and Larkins, 1989). In contrast to the H5-ELP and H5-hydrophobin PBs, H5-zein formed high density structures that were insoluble in non-reducing buffers, whereas H5-ELP and H5-hydrophobin fusion proteins could be extracted in 50 mM Tris, pH 8.0 (Phan et al., 2014).

The H5-zein bodies described herein were used as a delivery vehicle for a model vaccine antigen. IgG responses were elicited in all mice immunized with H5-zein but HI assays indicated the absence of neutralizing antibodies. Our results agree with previous parenteral immunization studies using monomeric hemagglutinin fused to ELP, which, in contrast to trimeric hemagglutinin, also did not induce neutralizing antibodies (Phan et al., 2013). Although the formation of PBs involves multiple cross-linking via intermolecular disulfide bonds, this type of multimerization may not be sufficient to support the specific oligomerization state that appears to be required for the formation of specific native epitopes that may confer a seroprotective immune response. Proper trimerization may be required to complete the folding of hemagglutinin monomers and to induce conformational effects necessary for full antigenicity and the induction of neutralizing antibodies (Magadan et al., 2013). The introduction of a trimerization signal in addition to the assembly sequence may therefore be beneficial, as reported for H5-ELP fusions (Phan et al., 2013).

One remarkable outcome of our experiments was that a comparable immune response was elicited in all mice despite the H5-zein concentration being 100 times lower than the concentration of soluble H5 in the control group, which was administered with a strong adjuvant. Interestingly, the administration of an adjuvant together with the H5-zein bodies did not promote a stronger immune response, suggesting that the addition of the zein portion itself mediates an adjuvant effect. This agrees with Whitehead et al. (Whitehead et al., 2014), who recently reported that the immunogenicity of a recombinant antigen was increased in the presence of Zera^®^, an assembly sequence that is very similar to the N-terminal part of γ-zein (Torrent et al., 2009b), and the immunogenicity of the fusion protein could not be enhanced further by the inclusion of Freund’s adjuvant. Similarly, the injection of synthetic zein microspheres that were loaded with ovalbumin resulted in higher IgG responses than the ‘free’ soluble protein (Hurtado-Lopez and Murdan, 2006a). This strongly supports the hypothesis that the zein N-terminal portion possesses intrinsic adjuvant activity, although we cannot exclude the possibility that the observed adjuvant effect was mediated by another component of the PBs. Joseph et al. reported that zein-induced PBs isolated from leaves contain additional proteins that are trapped during biogenesis (Joseph et al., 2012).

An adjuvant effect conferred by a polymer-forming protein domain is not unexpected, given its similarity to the strategy of attaching a carrier protein such as albumin or keyhole limpet hemocyanin to antigens with poor immunogenicity (Harris and Markl, 1999). By definition, an adjuvant is characterized by its ability to enhance the immunogenic efficacy of antigens in the same formulation. This can be achieved by increasing the half-life of an antigen, improving antigen delivery to its effector sites, or providing immunostimulatory signals to enhance the immune response. The observed adjuvant effect of zein particles may reflect one or more of several relevant properties. First, hydrophobic synthetic block copolymers have been shown to confer stronger adjuvant properties than hydrophilic polymers (Newman et al., 1998; Hunter, 2002). Accordingly, the N-terminal part of γ-zein is partially hydrophobic, favoring intermolecular and membrane interactions (Kogan et al., 2002). It has also been reported that particulate antigens are transported more efficiently to murine splenic follicular dendritic cells *in vivo* in the absence of prior immunity, making them more immunogenic than soluble antigens (Link et al., 2012). This may also be reflected by the superior immunogenicity of hemagglutinin-containing virus-like particles compared to soluble hemagglutinin, even in the absence of an adjuvant (Shoji et al., 2015). Also, repetitive antigen display, structural, or molecular mimicry of the virus, particle-size dependent tissue penetration and trafficking to lymphatics and Toll-like receptor activation are possible mechanisms. In repetitive antigen display the spatial organization of the antigens on the particle surface facilitates B-cell receptor (antibody) co-aggregation, triggering and activation. This can support the production of long-lived high-affinity neutralizing antibodies (Smith et al., 2013). Plant-derived PBs might also provide a specific spatial antigen organization favoring a successful repetitive antigen display. Alternatively, increased half life and stability of the zein fusions in the serum *in vivo* might be responsible for the enhanced immune response. The half-life of the antigen is likely to be extended due to encapsulation in the protein body. Indeed, pharmaceutical preparations encapsulated in zein particles *in vitro* remained in the blood for at least 24 h following intravenous delivery (Lai and Guo, 2011). Interestingly, the fusion of hemagglutinin to ELP repeats did not seem to increase immunogenicity although the propensity to form protein aggregates was confirmed *in planta* (Phan et al., 2013, 2014).

Zein has several favorable general characteristics as an adjuvant, i.e., it is stable at ambient temperatures and yet it is biodegradable, encouraging its use as a biopolymer for the coating and encapsulation of recombinant proteins such as erythropoietin (Bernstein et al., 1993). However, the potential immunogenic properties of zein must be taken into account (Hurtado-Lopez and Murdan, 2006a; Whitehead et al., 2014). We detected an immune response directed against γ-zein although the response was much weaker than that directed against H5, and when compared to the control group administered with H5 alone, the difference between the groups was not statistically significant. However, 30% of the mice injected with H5-zein showed an immune response above background levels. A significant immune response against the zein-like sequence Zera has been reported by (Whitehead et al., 2014), warranting further studies to assess the suitability of zein bodies as drug delivery vehicles for parenteral administration. Animal studies involving the injection of zein-coated erythropoietin (Bernstein et al., 1993) and ivermicin (Gong et al., 2011) did not indicate any adverse effects. Other storage proteins, including the wheat storage protein gliadin, have also been used as coatings to prepare various proteins and pharmaceuticals *in vitro*. GliSODin^®^ for example is an oral treatment for oxidative stress, in which superoxide dismutase (SOD) is coated with gliadin (Cloarec et al., 2007). Although gliadin protects SOD from digestion, this storage protein is also linked to the autoimmune disorder celiac disease (Chaptal, 1957). Even so, this product has received market approval for human use.

## Conclusion

Zein and similar plant storage proteins have long been investigated as carriers for pharmaceuticals including recombinant proteins. The direct encapsulation of pharmaceutical proteins in the production host is a simple approach that is less expensive than the production of synthetic microparticles. Our case study using a model vaccine antigen indicates that zein-induced PBs can be used as vaccine delivery vehicles that benefit from a value-added adjuvant effect, whereas the intrinsic immunogenicity of the zein component is low. The insertion of a trimerization signal fused to H5 will be tested to determine whether this leads to the assembly of structures that can elicit neutralizing antibodies against H5. It will also be of value to develop the *in planta* protein body encapsulation strategy for the production and delivery of further antigens, including candidates intended for oral application.

## Author Contributions

AH designed and carried out experiments, analyzed data, and wrote the manuscript. SM designed and carried out experiments and analyzed data. MT and EA carried out experiments, analyzed data, and contributed to the manuscript. HP, UG, and JL carried out experiments and analyzed data. UC and ES designed the study, analyzed data, and wrote the manuscript.

## Conflict of Interest Statement

The authors declare that the research was conducted in the absence of any commercial or financial relationships that could be construed as a potential conflict of interest.
